# The *Borrelia burgdorferi* c-di-GMP Binding Receptors, PlzA and PlzB, Are Functionally Distinct

**DOI:** 10.3389/fcimb.2018.00213

**Published:** 2018-07-11

**Authors:** Jessica L. Kostick-Dunn, Jerilyn R. Izac, John C. Freedman, Lee T. Szkotnicki, Lee D. Oliver, Richard T. Marconi

**Affiliations:** Department of Microbiology and Immunology, Virginia Commonwealth University Medical Center, Richmond, VA, United States

**Keywords:** *Borrelia*, Lyme disease, PlzB, cyclic-di-GMP, PlzA, PilZ

## Abstract

Cyclic-di-GMP (c-di-GMP) contributes to the regulation of processes required by the Lyme disease (LD) spirochetes to complete the tick-mammal enzootic cycle. Our understanding of the effector mechanisms of c-di-GMP in the *Borrelia* is evolving. While most LD spirochete isolates encode a single PilZ domain containing c-di-GMP receptor designated as PlzA, genome analyses have revealed that a subset encode a second PilZ domain protein (PlzB). The c-di-GMP binding potential of PlzB, and its role in LD spirochete biology, have not been investigated. To determine if PlzB binds c-di-GMP, *plzB* from *B. burgdorferi* isolate ZS7 was PCR amplified, cloned, and recombinant protein generated. PlzB bound c-di-GMP but not other nucleotides, indicating a specific binding interaction. To determine if PlzA and PlzB are functionally synonymous, a series of allelic-exchange gene deletion and *cis*-complemented strains were generated in the *B. burgdorferi* B31 background. *B. burgdorferi* B31-Δ*plzA* was competent to infect *Ixodes scapularis* larvae but not mice when delivered by either needle or tick feeding. *B. burgdorferi* B31-Δ*plzA* also displayed an atypical motility phenotype. Complementation in *cis* of *B. burgdorferi* B31-Δ*plzA* with *plzA* (B31-*plzA* KI) restored wild-type (wt) phenotype. However, a strain complemented in *cis* with *plzB* (B31-*plzB* KI) did not. The data presented here are consistent with an earlier study that demonstrated that PlzA plays an essential role in spirochete survival in the mammalian environment. We add to our understanding of the c-di-GMP regulatory network by demonstrating that while PlzB binds c-di-GMP, it is not functionally synonymous with PlzA. The absence of *plzB* from most strains suggests that it is not required for survival. One possibility is that cells that harbor both PlzA and PlzB might have enhanced biological fitness or increased virulence.

## Introduction

*Borrelia burgdorferi, B. garinii, B. bavariensis*, and *B. afzelii* are the main causative agents of Lyme disease (LD) in North America and Europe (Burgdorfer et al., 1982). Henceforth, we collectively refer to all *Borrelia* species that cause LD as the LD spirochetes. Note that the LD spirochetes have recently been assigned to a new genus, *Borreliella* (Adeolu and Gupta, 2014). The use of this new designation is voluntary. In this report, we continue to use the *Borrelia* genus designation. The LD spirochetes are host-dependent extracellular pathogens that are maintained in nature by cycling between *Ixodes* ticks and diverse mammalian reservoirs (Barbour and Hayes, 1986). To successfully complete the enzootic cycle, rapid adaptation to environmental conditions is essential. c-di-GMP is a key contributor to adaptive responses (Ryjenkov et al., 2005; Rogers et al., 2009; Sultan et al., 2010; Kostick et al., 2011; Pitzer et al., 2011). In *B. burgdorferi*, c-di-GMP levels are regulated by the opposing activities of Rrp1 [a GGDEF domain containing diguanylate cyclase (DGC)] and PdeA and PdeB [EAL and HD-GYP domain containing phosphodiesterases (PDE)] (Galperin et al., 2001; Ryjenkov et al., 2005; Rogers et al., 2009; Sultan et al., 2010). Orthologs of Rrp1, PdeA, and PdeB are encoded by the genomes of all LD and tick-borne relapsing fever spirochete species. The only demonstrated functional activity of Rrp1 is c-di-GMP production. Studies of *B. burgdorferi rrp1* deletion mutants have revealed that Rrp1, and by extension c-di-GMP, directly or indirectly regulates a significant proportion of the *B. burgdorferi* transcriptome (Rogers et al., 2009; Caimano et al., 2015). C-di-GMP levels influence motility, N-acetyl glucosamine directed chemotaxis, glycerol metabolism, chitobiose utilization, and several other metabolic pathways (Rogers et al., 2009; Kostick et al., 2011; Sze et al., 2013; Caimano et al., 2015, 2016). Interestingly, c-di-GMP is not required by the LD spirochetes to infect mammals but it is essential for survival in ticks (Rogers et al., 2009; Kostick et al., 2011; Caimano et al., 2015, 2016).

Proteins harboring C-terminally located PilZ domains are among the primary receptors and effector partners for c-di-GMP (Amikam and Galperin, 2006). *B. burgdorferi* B31, and most other LD spirochete isolates, encode and produce a single c-di-GMP receptor designated as PlzA (Freedman et al., 2009). PlzA is a 29.6 kDa (261 aa) stand-alone PilZ domain protein (i.e., lacks other identifiable conserved functional domains) that is chromosomally encoded (*B. burgdorferi* B31 ORF designation, BB0733). The tick-borne relapsing fever spirochetes encode a related c-di-GMP receptor designated as PlzC that is also encoded by a linear chromosome carried gene (Freedman et al., 2009; Mallory et al., 2016). PlzA has 69% amino acid identity to PlzC. Bacterial PilZ domains are identified by two signature motifis; RxxxR and DzSxxG (Amikam and Galperin, 2006). In the *Borrelia*, these consensus motifs have been further defined as RIHER and DzSYGG (Mallory et al., 2016). The requirement for specific residues within these motifs in c-di-GMP binding has been revealed through site-directed amino acid substitution mutagenesis (Freedman et al., 2009; Mallory et al., 2016). PlzA and PlzC are monomeric in both their apo and holo forms and bind c-di-GMP with a 1:1 stoichiometry (Mallory et al., 2016). C-di-GMP binding to PlzA and PlzC triggers structural rearrangements that may serve as a functional switch (Mallory et al., 2016). While the full range of regulatory activities mediated by PlzA and PlzC are not defined, studies of deletion mutants have demonstrated that PlzA is linked to motility (Pitzer et al., 2011). In addition, PlzA influences the regulatory activity of BosR, a DNA binding protein in the Fur/PerR family that is required for survival in mammals but not ticks (Seshu et al., 2004; Hyde et al., 2006, 2007, 2009; Ouyang et al., 2011; He et al., 2014).

A gene encoding a second PilZ domain-containing protein designated as PlzB has been identified on a linear plasmid of 28 kb in a subset of LD spirochete isolates (Freedman et al., 2009). The function and biological significance of PlzB has not been investigated. *plzB* encodes a putative 30 kDa protein with ~64 and 53% amino acid identity to PlzA and PlzC, respectively. In this report, the ability of PlzB to bind c-di-GMP and the functional relationship between PlzA and PlzB were assessed. In summary, PlzB binds c-di-GMP in a highly specific manner. Consistent with an earlier report (Sultan et al., 2010), deletion of *plzA* results in an aberrant motility pattern and eliminates or attenuates infectivity of *B. burgdorferi* B31 in mice. The ability to infect larval stage *Ixodes scapularis* ticks was unaffected. While PlzB bound c-di-GMP, *cis*-complementation of a *plzA* deficient strain with *plzB* did not restore wt (wild type) phenotype. The studies presented within indicate that while PlzB is competent to serve as a receptor for c-di-GMP, PlzA, and PlzB are not functionally synonymous.

## Materials and methods

### Bacterial strains and cultivation conditions

*Borrelia* isolates were cultivated in BSK media (33°C; 5% CO_2_) prepared using bovine serum albumin from Gemini Bio-Products, Inc. (lot #C54) as previously described (Samuels et al., 1994). All genetically manipulated strains were initially cultivated in BSK media or BSK semi-solid media plates containing 75 μg/mL streptomycin or 75 μg/mL kanamycin, as appropriate. BL21 (DE3) and TOP10 *E. coli* cells were cultivated in LB Broth, Lennox, or LB agar (Fisher BioReagents) containing 100 μg/mL ampicillin at temperatures detailed below.

### Generation and expression of recombinant proteins

*plzA* and *rrp1* were PCR amplified from *B. burgdorferi* B31 using standard methods. *plzB* was amplified from *B. burgdorferi* strain ZS7. The primers were designed to include 5′ *Bam*HI or 3′ *Sal*I restriction sites as required. The amplicons were digested with the appropriate restriction enzyme, ligated into the multiple cloning site of pCR2.1-TOPO, transformed into TOP10 *E. coli* cells (Invitrogen), and colonies with the appropriate plasmid inserts identified by PCR. The plasmids were propagated and subcloned into pMAL-c4x (New England BioLabs) via the *Bam*HI and *Sal*I restriction sites to yield pMAL-c4x*plzA*, pMAL-c4x*plzB*, and pMAL-c4x*rrp1*. The plasmids were purified and transformed into BL21 (DE3) *E. coli* cells (Novagen) for protein expression. Cells were grown at 37°C to an OD_600_ of 0.6, shifted to 23°C, and protein expression induced with isopropyl-beta-D-1-thiogalactopyranoside (IPTG; 1 mM; 3 h). Cells were harvested by centrifugation, treated with lysozyme (1 mg/mL), sonicated, and soluble protein purified by amylose affinity chromatography as instructed by the supplier (New England BioLabs). It is worth noting that in subsequent work, each protein was successfully produced with N-terminal Hexa-His tags using the pET-45b (+) vector.

### Synthesis of radiolabeled c-di-GMP

C-di-GMP was enzymatically synthesized using recombinant WspR as previously described (Freedman et al., 2009). In brief, the *wspR* gene sequence from *Pseudomonas aeruginosa* FRG-1 was PCR amplified and inserted into a cloning vector to produce recombinant protein. To activate the DGC activity of r-WspR, WspR was incubated with acetyl phosphate. Phosphorylated WspR was incubated with GTP [α- ^32^P] or GTP to generate labeled or cold (unlabeled) c-di-GMP, respectively. C-di-GMP concentrations and purity were assessed using reverse phase FPLC with GTP and unlabeled c-di-GMP served as elution standards. Nucleotides were detected at a wavelength of 254 nm.

### PlzA and PlzB c-di-GMP binding assays

C-di-GMP binding assays were carried out as previously described (Freedman et al., 2009). In brief, recombinant PlzA and PlzB were spotted onto nitrocellulose membranes and air dried. Rrp1 served as a negative control. The membranes were incubated with [^32^P]c-di-GMP (2 nM) alone, or in combination with 750 nM of c-di-GMP, GTP, GMP, ATP, cGMP, or cAMP (Sigma; PBS with 1% non-fat dried milk; 2 h; room temperature), washed twice with PBS (10 min) wrapped in cellophane and exposed to film overnight with intensifying screens at −80°C.

### Allelic exchange mutagenesis

Allelic replacement of *plzA* was performed using methods developed by Samuels et al. (1994). One kb of DNA upstream and downstream of *plzA* were PCR amplified in separate reactions and cloned into the pCR2.1-TOPO vector to yield pCR2.1-UP and pCR2.1-DN, respectively. The upstream (UP) and downstream (DN) 1 kb fragments possessed *Aat*II and *Age*I restriction sites at the appropriate end to allow for subsequent transfer into other plasmids. The DN fragment was excised from the pCR2.1-DN with *Aat*II and *Age*I, ligated into pCR2.1-UP vector to generate pCR2.1-UPDN. A spectinomycin resistance cassette, derived from pKFSS1-*Aat*II (Frank et al., 2003), was inserted into the *Aat*II site at the junction between the UP and DN fragments to yield pCR2.1Δ*plzA-spec*^*R*^. The plasmid was linearized with *Msc*I and *Sca*I and electroporated into infectious *B. burgdorferi* B31-5A4 to generate the B31-Δ*plzA* mutant. Electroporation conditions were as previously described (Samuels et al., 1994). Selection was achieved by supplementing BSK with 75 μg ml^−1^ streptomycin. Clonal populations were obtained by sub-surface plating (Sung et al., 2001). The plasmid content of each clone was determined by PCR utilizing plasmid-specific primers (Labandeira-Rey et al., 2003) with some modifications (Rogers and Marconi, 2007).

*Cis*-complementation of *plzA* was obtained by replacement of the spectinomycin cassette with *plzA* and downstream kanamycin resistance cassette. A PCR product, containing the *plzA* upstream sequence, *plzA* ORF, and 3′ flanking *Aat*II and *Asc*I sites, was TOPO cloned into pCR2.1 to generate pCR2.1*plzA*-UP. A kanamycin resistance cassette was PCR amplified from pBSV2 (Stewart et al., 2001) with a 5′ *Aat*II site and joined to a PCR product derived from the 1,000 bp of native sequence located immediately downstream of B31 *plzA* with a 3′ *Asc*I site. The kanamycin-DN fusion was TOPO cloned into pCR2.1, digested and ligated into pCR2.1*plzA*-UP to create pCR2.1 *plzA-kan*. The plasmid was linearized with *Sca*I and *Msc*I and electroporated into B31-Δ*plzA* to yield the *cis-*complemented strain B31-*plzA* KI. Selection was achieved with 75 μg ml^−1^ kanamycin. Clonal populations were isolated from semi-solid BSK media plates and screened for plasmid profile as described above.

### *plzB* allelic replacement of *plzA*

Replacement of *plzA* with *plzB* was achieved by allelic exchange mutagenesis. The upstream 1 kb region of *plzA* was PCR amplified to include the *plzA* ribosome-binding site (RBS) and cloned into pCR2.1-TOPO to generate plasmid pCR2.1*plzA*-UP2. The 1kb DN fragment was excised from pCR2.1*plzA*-DN with *Aat*II and *Age*I and ligated into pCR2.1*plzA*-UP2 to yield pCR2.1*plzA*-UPDN2. *plzB* was PCR amplified from *B. burgdorferi* ZS7 with primers that introduced a 5′ *Aat*II site and a 3′ overlap with the P_*flgB*_*kan*^*R*^ cassette. The P_*flgB*_*kan*^*R*^ cassette was amplified from pBSV2 (Stewart et al., 2001) to possess a 5′ overlap with the 3′ *plzB* ORF and a 3′ *Age*I restriction site. A *plzB*- P_*flgB*_*kan*^*R*^ fusion was created by overlap extension PCR, then digested with *Aat*II and *Age*I, and ligated into pCR2.1*plzA*-UPDN2 to yield pCR2.1*plzB*-kan. The plasmid was linearized with *Sca*I and *Nco*I, electroporated into *B. burgdorferi* B31-5A4, and transformants selected by cultivation in media containing 75 μg ml^−1^ kanamycin.

### SDS-PAGE, immunoblot and ELISA analyses

Cell lysates were fractionated using 12.5% Criterion Precast Gels (BioRad) and transferred to PVDF membranes by electroblotting. The membranes were incubated with blocking buffer (1x PBS, 0.2% Tween-20, 5% non-fat dry milk) and screened with serum from mice infected or inoculated with each strain at a dilution of 1:1,000. Antibody binding was detected using horseradish peroxidase (HRP)-conjugated secondary antibodies (Pierce; 1:40,000) and SuperSignal West Pico chemiluminescence substrate (Pierce). For ELISAs, 96-well plates (Costar 3590) were coated with B31-5A4 spirochetes (0.1 OD_600_ of ml^−1^) in carbonate buffer (100 mM NaHCO_3_, pH 9.6). Blocking buffer (1% BSA in PBS-T) was added to each well (2 h; room temperature; with shaking). The desired sera were then added in 3-fold serial dilutions (1:50-1:109,350; in triplicate). Plates were washed 3 times with PBS-T and bound IgG was detected with HRP-conjugated goat-anti-mouse IgG antiserum (1:20,000) with ABTS substrate. The plates were read at OD_405_. The absorbance values obtained from the pre-bleed sera were considered to be baseline and these values were subtracted prior to titer calculation. Titers were calculated as previously described (Earnhart et al., 2010). Statistical analysis was performed using a one-way ANOVA.

### RNA isolation and reverse transcriptase polymerase chain reaction (RT-PCR)

RNA was isolated from *Borrelia* cells using the RNeasy Midi kit (Qiagen). Residual DNA was removed by treatment with DNase I (Invitrogen). cDNA was produced using the Superscript III First Strand cDNA Synthesis kit (Invitrogen), 50 ng of random hexamer primers, and 1 μg total RNA. RT-PCR was performed with SYBR green PCR Master Mix (Applied Biosystems) and a CFX96 Real Time System (BioRad) with the following cycle parameters: 1 cycle of 10 min at 95°C followed by 40 cycles of 10 s at 94°C, 30 s at 50°C, and 30 s at 72°C.

### Motility analyses

Rotational (wave propagation) and translational (directional movement) motility of spirochetes in BSK with or without 1% carboxy methylcellulose (CMC) was assessed by dark-field microscopy and differential interference contrast (DIC) microscopy. CMC was added to the culture media to increase viscosity. Motility and flex rates were determined using Slidebook 5 (Intelligent Imaging Innovations) motion-tracking software and represented the average of 20 motility tracking analyses per *Borrelia* strain.

### Swarm plate and capillary tube chemotaxis assays

Swarm assays (migration of spirochetes through semi-solid media) were conducted as previously described (Kostick et al., 2011). Cells were introduced into BSK-H semi-solid media plates containing 0.35% (weight/vol) SeaKem GTG agarose by insertion into a depression in the media generated by coring with a 1 ml micropipette tip. 5 × 10^5^ spirochetes (5 μl of a 1:10 BSK-H dilution in dPBS) were introduced into each depression. All assays were performed in triplicate. Plates were incubated at 33°C and the diameter of the visible expanding colony assessed at 2, 4, and 6 days. Capillary chemotaxis assays were performed as previously described (Kostick et al., 2011) using 0.1M N-acetyl-D-glucosamine as the chemoattractant. One-way ANOVA statistical analysis was performed on the means from 3 separate experiments.

### Murine infection, seroconversion, and cultivation of spirochetes

The ability of each strain to infect C3H-HeJ mice (Jackson Labs) was determined using 10^4^ spirochetes (in 100 μl BSK-H complete media) delivered by subcutaneous needle inoculation between the shoulder blades. Four weeks post-infection, mice were sacrificed, blood was obtained by cardiac puncture and tissue and organs collected. Serum was harvested using standard methods and seroconversion of each individual mouse assessed by ELISA and immunoblot as detailed above. To cultivate spirochetes from the mice, sections of tissues and organs were placed in BSK media supplemented with an antimicrobial cocktail consisting of phosphomycin, rifampicin, and amphotericin B (Sigma) and growth monitored using dark-field microscopy. All animal experiments were conducted following the Guide for the Care and Use of Laboratory Animals (8th edition) and in accordance with protocols peer reviewed and approved by VCU Institutional Animal Care and Use Committees.

### Infection of ticks by immersion in cultures

Infection free-*I. scapularis* larvae (Oklahoma State University Tick Rearing Facility) were infected by immersion in *B. burgdorferi* cultures (10^8^ spirochetes ml^−1^; 33°C; 2 h) as previously described (Policastro and Schwan, 2003). Ticks were recovered, washed, dried, maintained for 2 days and then fed on naive C3H-HeJ mice. One week after completion of tick feeding, DNA was isolated from the ticks using the DNeasy Blood and Tissue Kit (Qiagen). Randomly selected ticks were analyzed for spirochetes by PCR (*flaB*). Normalization against tick DNA was achieved using RIB-3 and RIB-4 primers (Zahler et al., 1995) as previously described (Kostick et al., 2011). PCR results were scored as plus or minus.

## Results

### PlzB binds cyclic di-GMP with high specificity

**Figure 1:**
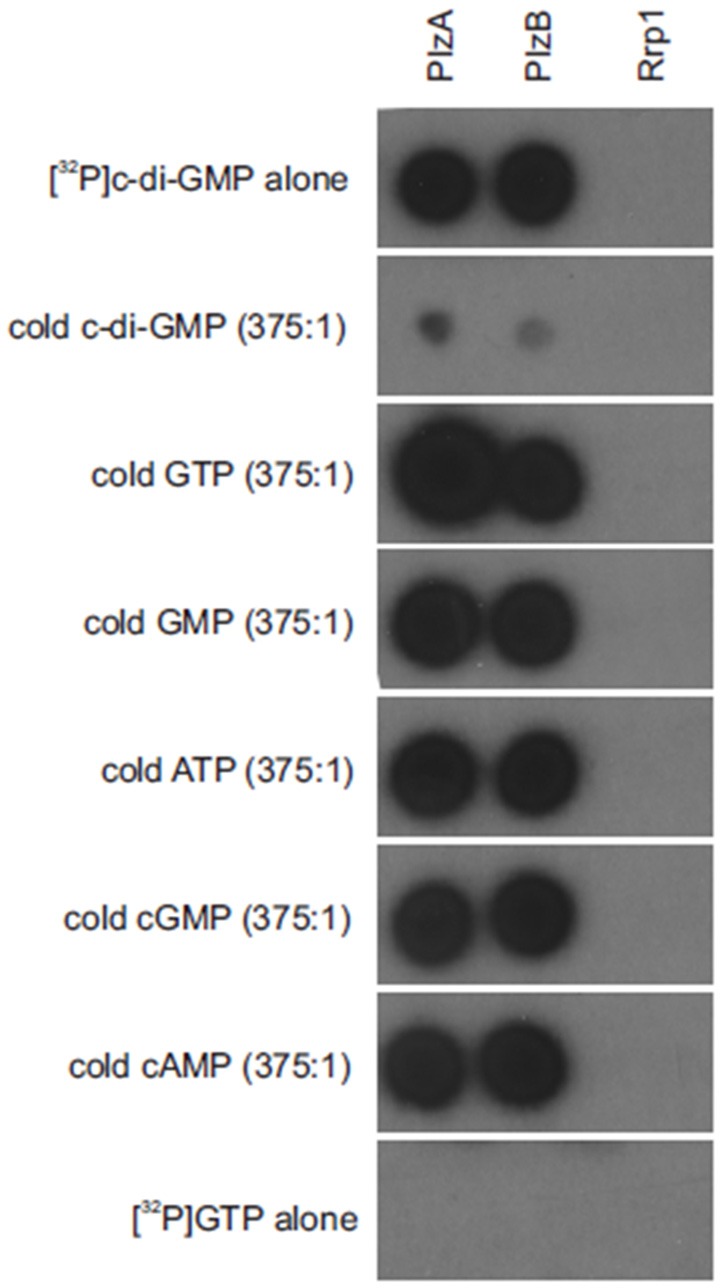
*Borrelia burgdorferi* PlzB binds to c-di-GMP with high specificity. Equal amounts (0.25 μg) of recombinant PlzB, PlzA (positive control), and Rrp1(negative control) were spotted onto a series of nitrocellulose membrane strips and incubated with [^32^P]c-di-GMP alone (top; 2 nM) or [^32^P]c-di-GMP plus a potential competing unlabeled nucleotide (750 nM; 375:1 competitor to [^32^P]c-di-GMP molar ratio). In the bottom, labeled [^32^P] GTP alone was incubated with the membrane as a control for possible non-specific radiographic signal. After incubation, all membranes were washed identically and exposed to film. All methods are as detailed in the text.

(^32^P) c-di-GMP binding to PlzB, PlzA (positive control) and Rrp1 (negative control) was assessed using a membrane overlay assay (Freedman et al., 2009). The purity of the radiolabeled c-di-GMP generated using WspR was verified through reverse phase liquid chromatography (Freedman et al., 2009). C-di-GMP bound to PlzB and PlzA but not to Rrp1. Binding was inhibited by unlabeled c-di-GMP but not by GTP, ATP, cAMP, or cGMP (Figure 1). It can be concluded that recombinant PlzB can serve as a receptor for c-di-GMP.

### Characterization of genetically modified strains of *B. burgdorferi*

**Figure 2:**
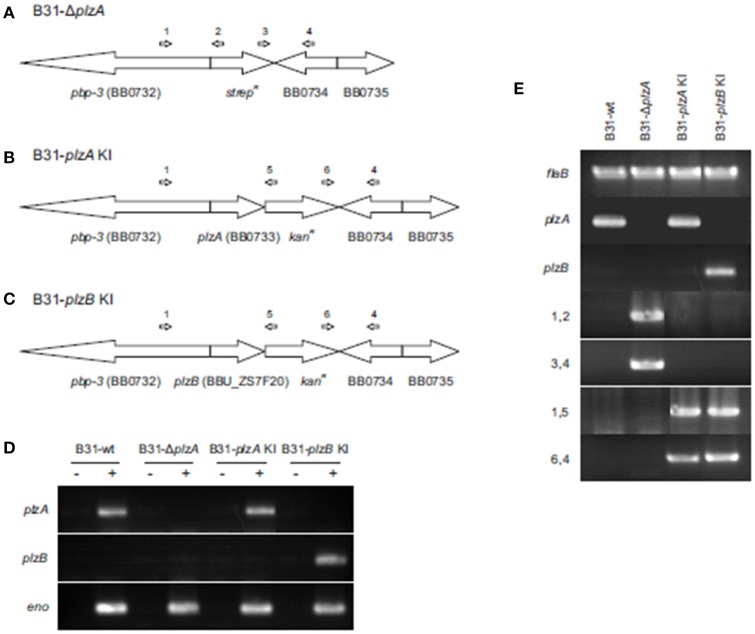
Generation and confirmation of genetic constructs. **(A)** Depicts in schematic form the organization of the *plzA* locus of the *B. burgdorferi* B31 linear chromosome after replacement of *plzA* with a *strep*^*R*^ cassette. **(B,C)** Depict the gene organization of the same region of the chromosome after *cis-*complementation or “knock in” (KI) with *plzA* or *plzB*, respectively. Note that the intergenic regions between are not indicated in the schematics. The ORF designations originally assigned to the *B. burgdorferi* B31 genome are indicated. The general location of hybridization sites for primers used to verify the intended allelic exchange events are indicated above each schematic with the PCR results presented in **(E)**. Amplification of the *flaB* gene served as a positive control for PCR. **(D)** Shows the results of RT-PCR analyses conducted to assess transcription of each gene. Amplification of the enolase gene served as a positive control for RT-PCR. The plus and minus signs indicate the inclusion or absence of reverse transcriptase in the reactions. All primers used in this study are listed in Table 1. Note that the PCR gel images are composites of images from independent gels that were cropped and aligned for reader convenience.

*plzA* was deleted from the *B. burgdorferi* B31-5A4 infectious clone (B31-wt) and replaced with a spectinomycin/streptomycin resistance cassette (*strep*^*R*^) to yield B31-Δ*plzA* (Figure 2A). A *cis*-complemented *plzA* strain was generated by replacing the *strep*^*R*^ cassette of B31-Δ*plzA* with *plzA* and a downstream kanamycin resistance cassette (*kan*^*R*^) to yield B31-*plzA* KI (KI indicating “knock-in”) (Figure 2B). To substitute *plzA* with *plzB*, the *strep*^*R*^ cassette of B31-Δ*plzA* was replaced with *plzB-kan*^*R*^ to yield B31-*plzB* KI (Figure 2C). Clonal populations of the mutated strains were obtained by subsurface plating and proper deletion/insertion was verified by PCR (Figure 2E, Table 1). Plasmid specific PCR analyses of the clonal populations identified several that possess the full complement of plasmids carried by the B31-wt parental strain (21 circular and linear plasmids; data not shown). Individual clones of each strain were selected for further analysis.

**Table 1:** Oligonucleotides used in this study.

**Oligonucleotide**	**Sequence (5^′^ → 3^′^)**
**General primers**	
PlzA KO >1 kb upstr F(1)	GCTATCATTGCTCCTTCAGGCTGTGC
PlzA KO >1 kb downstr R(2)	CCTTTAATAGTACATGTTGATACGG
aad1-5^′^-R(3)	TCCTTGAAGCTCGGGTATTA
pKFSS1-3^′^F(4)	GGCGAGATCACCAAGGTAGTC
kan-5^′^-R(5)	CAGCATCCATGTTGGAATTTAATCGC
kan-3^′^-F(6)	GATATGAATAAATTGCAGTTTCATTTG
flab-F	CAGGTAACGGCACATATTCAGATGC
flab-R	CTTGGTTTGCTCCAACATGAACTC
PlzA-F-LIC	GACGACGACAAGATTTTGTTTAGTATTTTTATATTCAAAAAAAGGAGAAAG
PlzA-R-LIC	GAGGAGAAGCCCGGTTTAATTGAAATAATCATGGATCAACATAGATAC
Rrp1-F-LIC	GACGACGACAAGATGGAAATGATAATTAAAGATAAAGC
Rrp1-R-LIC	GAGGAGAAGCCCGGTTTAATATCTAAACTGATTTCTTCCAG
***plzA*****deletion vector creation**	
PlzA-UP-F	CATTTGATACAACTTGGTTTAAAACTG
PlzA-UP-R	ACCGGTCTAGACGTCAGCTTCAAATTGTTTTAAACAGTTTTACACCGGTCTAGAGCT
PlzA-DN-F	GACGTCTCAAAGGATTGAAATTTTTCTTATGTGATTATG
PlzA-DN-R	ACCGGTCAGAATATATTCCCAAAAGTGCCC
Spec/strep-F	TGATTTGCTGGTTACGGTGA
Spec/strep-R	ATTTGCCGACTACCTTGGTG
***plzA*****KI construction**	
PlzA-KI-F	GACGTCTTGTTTAGTATTTTTATATTCAAAAAAAGGAGAAAG
PlzA-KI-R	CCTTGAAGCTCGGGTATTAGTTAATTGAAATAATCATGGATCAACATAGTATAC
PflgB-aphI-T7(PlzA)KI-F	GTATACTATGTTTAGCCATGATTATTTCAATTAACTAATACCCGAGCTTCAAGG
PflgB-aphI-T7-KI-R	GACGTCCAGATCCGGATATAGTTCCTCCTTTC
**B31-plzB-KI generation**	
PlzB-KI-UP-F	CATTTGATACAACTTGGTTTAAAACTG
PlzB-KI-UP-R	TATCACACCGGTGACGTCAAACCTCTCCTTTCTCCTTTTTTTG
PlzB-KI-F	GACGTCATGGCAGTATCATCTAAAAAGATAAGAGAG
PlzB-KI-R	CTTCCTTGAAGCTCGGGTAGTCAGTCTTCAAAAAAATTAAAATAATTATG
Kan KI-F	TATCACCCCGGGCTAATACCCGAGCTTCAAGGAAGA
Kan KI-R	TATCACGACGTCTTAGAAAAACTCATCGAGCATCAAATGAAAC
**RT-PCR**	
PlzA-F-RT	CTGATAAAGCTTTTATCAAGTTTAATGGAG
PlzA-R-RT	AGCGCAAAAACCTTTCCGCT
PlzB-F-RT	TAGTATACTCAAGCGGGATATTACTC
PlzB-R-RT	GGAATCCTTGATGAAGACATGG
Eno(BB0337)F-RT	GCTTGAACTTGATGGCACCCCTAC
Eno(BB0337)R-RT	GTACGCTCCAAGATATTGATAAGG
**pMAL-c4x PlzB**	
PlzB pMAL-BamHI-F	GGATCCATGGCAGTATCATCTAAAAAGATAAGAGAGTATAGAAA
PlzB pMAL-SalI-R	GTCGACTCAGTCTTCAAAAAAATTAAAATAATTATGGATTATCATAGTATACTCAA

Transcription of *plzA* or *plzB* was assessed by RT-PCR and as expected, *plzA* mRNA was detected in B31-wt and B31-*plzA* KI but not in B31-Δ*plzA* and B31 *plzB-*KI. *plzB* transcript was detected in B31 *plzB* KI but not in the other strains (Figure 2D). To determine if the process of genetic manipulation had an impact on *in vitro* growth, standard growth curves were generated at 27°, 33°, and 37°C (data not shown) using previously established methods (Earnhart et al., 2010). Deletion of *plzA*, complementation with *plzA* or *plzB*, or the introduction of *kan*^*R*^*, spec*^*R*^, or *amp*^*R*^ antibiotic resistance cassettes had no effect on growth rate at any temperature.

### The motility defects in a *plzA* deletion mutant can be restored by complementation with *plzA* but not *plzB*

**Figure 3:**
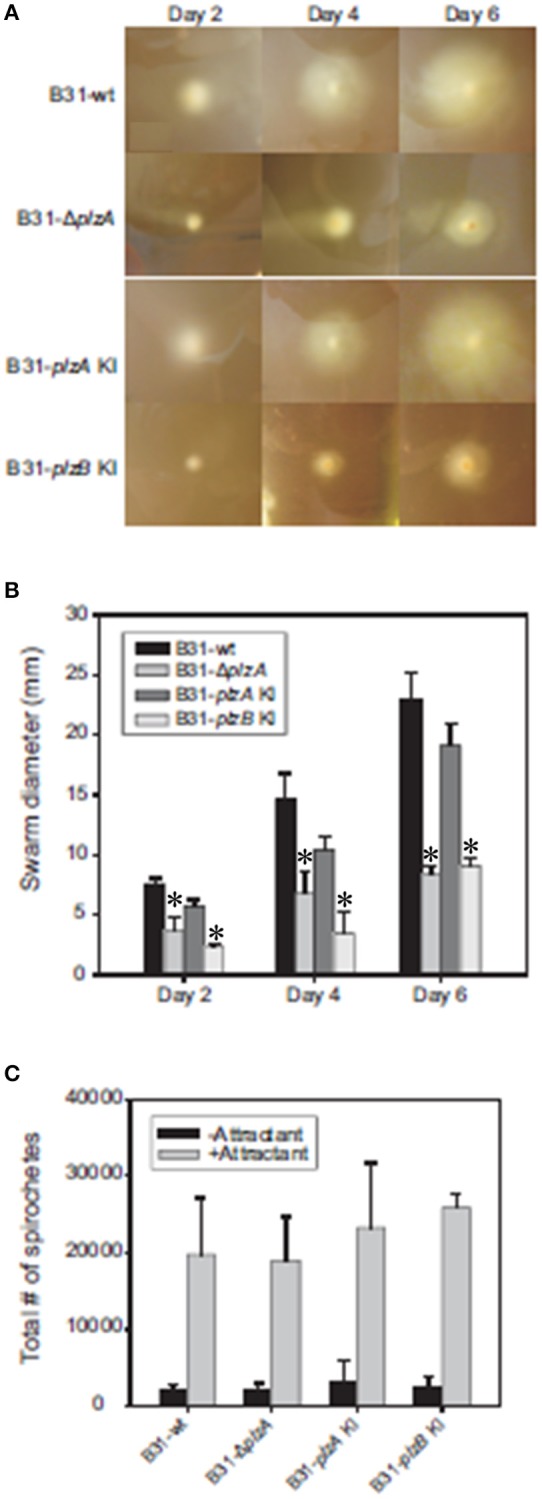
Motility and chemotaxis analyses. *B. burgdorferi* B31-wt, B31-Δ*plzA*, B31-*plzA* KI, and B31-*plzB* KI were evaluated for motility using a “plate swarming” assay as detailed in the text. Photographs of representative plates taken 2, 4, and 6 days after inoculation of the plates are shown in **(A)**. The diameters of each expanding zone of growth was determined and are presented as bar graphs in **(B)**. In **(C)**, the results of capillary tube-based chemotaxis assays using N-acetyl glutamic acid as the chemoattractant are shown. The assays were conducted as detailed in the text and statistical significance was assessed using the One-Way ANOVA. ^*^Statistical significance was assessed relative to wild type (*p* < 0.05).

Motility patterns of cells growing in BSK with 1% CMC were assessed using dark-field and DIC microscopy. While all strains displayed similar rotational motility (wave propagation) as assessed by dark-field microscopy, the rate of translational motility (directional movement) and flexing differed (as determined using DIC microscopy). B31-Δ*plzA* and B31-*plzB* KI had reduced translational motility and flex frequency, whereas the B31-*plzA* KI complemented strain displayed wt motility patterns (data are summarized in Table 2). Motility was assessed using swarm assays (Kostick et al., 2011). Colony diameters for the B31-Δ*plzA* and B31-*plzB* KI strains were reduced by ~60% in comparison to B31-wt (*p* < 0.05) (Figures 3A,B). Complementation with *plzA*, but not *plzB*, restored normal colony size (swarm diameter). The chemotactic responses of each strain to N-acetyl-D-glucosamine (NAG) were also assessed. Deletion of *plzA* had no effect on chemotactic responses to NAG (Figure 3C). Note that other potential chemoattractants were not tested. The data indicate that PlzA plays a direct or indirect role in translational motility, but not rotational motility, and that this specific PlzA function is not complemented by PlzB.

**Table 2:** Motility patterns and observations.

**Strain**	**BSK-H**	**1% Methylcellulose**
B31-wt	Normal motility	Translational motion: runs, stops/flexes, reverses Avg run velocity: 4.158 ± 0.779 μm/s Flexes/sec: 0.0783 ± 0.0313
B31-Δ*plzA*	Normal motility	Translational motion: runs with very infrequent stops/flexes Avg run velocity: 1.650 ± 0.734 μm/s (^*^p < 0.01) Flex events/sec: 0.004 ± 0.012 (^*^p < 0.01)
B31-*plzA* KI	Normal motility	Translational motion: runs, stops/flexes, reverses Avg run velocity: 4.1706 ± 0.8714 μm/s Flex events/: 0.1024 ± 0.0541
B31-*plzB* KI	Normal motility	Translational motion: runs with infrequent stops or flex events Avg run velocity: 1.5793 ± 0.7369 μm/s (^*^p < 0.01) Flexes/sec: 0.0078 ± 0.0109 (^*^p < 0.01)

* *p < 0.01*.

### Assessment of the ability of *B. burgdorferi* B31 wild type, *plzA* gene deletion and *plzA/B cis*-complemented strains to infect mice

**Figure 4:**
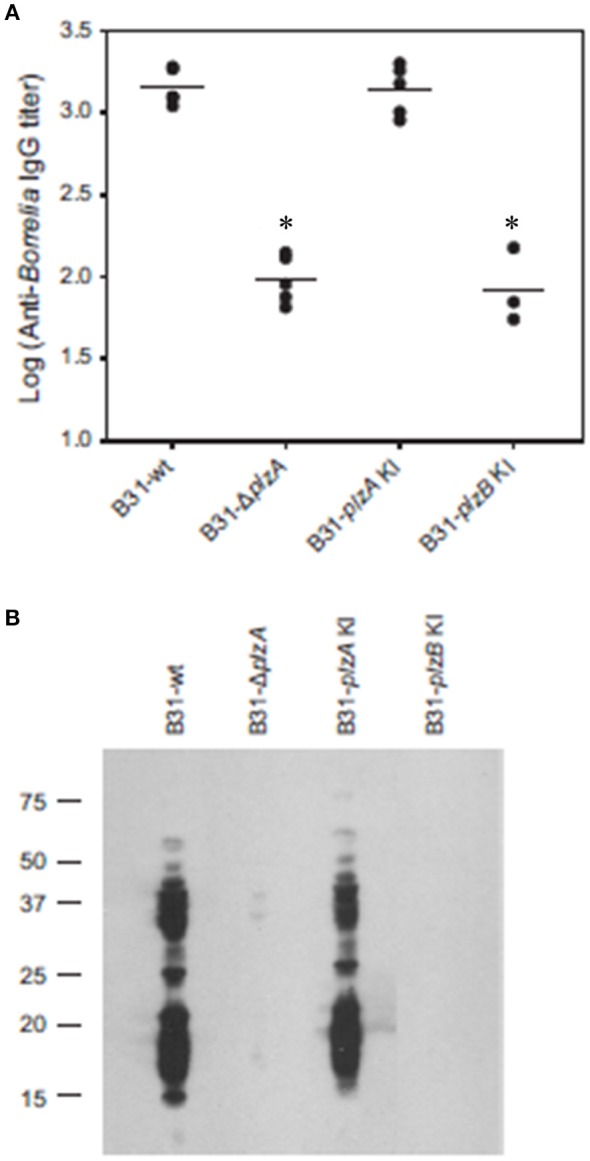
PlzB does not restore infectivity to *plzA* gene deletion mutant delivered by needle inoculation. C3H-HeJ mice were inoculated by subcutaneous needle injection with 10^4^ spirochetes of each strain investigated. Serum was collected from each mouse 4 weeks post-inoculation and anti*-B. burgdorferi* IgG titer was determined by ELISA (endpoint dilution) using whole cell lysate as the detecting antigen **(A)**. **(B)** Shows the results of immunoblot analyses. *B. burgdorferi* B31 served as the immobilized antigen. For the immunoblot analyses, serum samples from each experimental group were pooled. All methods were conducted as detailed in the text and statistical significance was assessed using the One-Way ANOVA. ^*^Statistical significance was assessed relative to wild type (*p* < 0.05).

Each strain was delivered to five C3H-HeJ mice by subcutaneous needle inoculation. Tissue and organ biopsies (bladder, heart, and skin) were harvested after 4 weeks, sections were placed in BSK and spirochete growth monitored. All biopsy samples (*n* = 15; one sample from heart, bladder and tissue from each mouse) yielded positive cultures except those obtained from mice that had been inoculated with B31-Δ*plzA* and B31-*plzB* KI (Table 3). All mice that yielded positive cultures were also seropositive as assessed by ELISA (Figure 4A). The IgG titers in mice infected with B31-wt and B31-*plzA* KI were similar while significantly lower titers were measured in B31-Δ*plzA* and B31*plzB* KI (*p* < 0.01). Immunoblot analyses using *B. burgdorferi* B31whole cell lysate as the detecting antigen yielded results that were consistent with the ELISA and cultivation data (Figure 4B). As expected, the immunoblot analyses revealed that a wide range of proteins were recognized by antibody elicited by infection.

**Table 3:** Murine infection study.

**Strain**	**No. of spirochete positive cultures from each tissue or organ biopsy**			
	**Bladder**	**Heart**	**Skin**	**No. of culture positive/tested**
B31-wt	5/5	5/5	5/5	5/5
B31-Δ*plzA*	0/5	0/5	0/5	0/5
B31-*plzA* KI	5/5	5/5	5/5	5/5
B31-*plzB* KI	0/3	0/3	0/3	0/3

**Figure 5:**
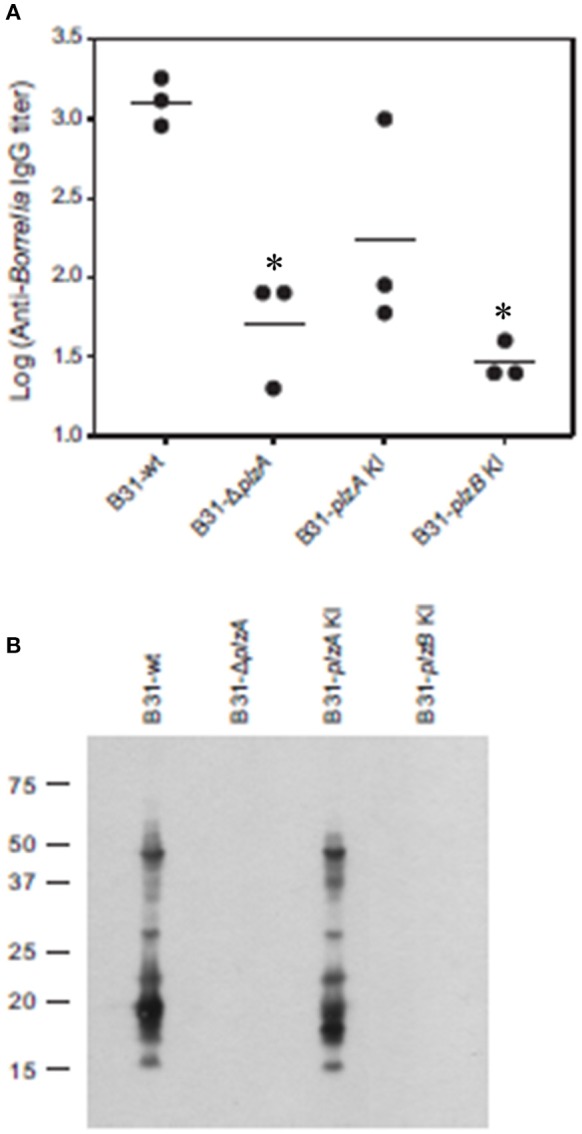
Tick to mouse spirochete transmission analyses. *I. scapularis* larvae were infected with each strain by immersion in actively growing cultures. After verifying infection, the infected larvae were fed on naive C3H-HeJ mice. Serum was collected from each mouse 4 weeks post-inoculation and anti*-B. burgdorferi* IgG titer was determined by ELISA (endpoint dilution) using whole cell lysate as the detecting antigen **(A)**. **(B)** Shows the results of immunoblot analyses. *B. burgdorferi* B31 served as the immobilized antigen. For the immunoblot analyses, serum samples from each experimental group were pooled. All methods were conducted as detailed in the text and statistical significance was assessed using the One-Way ANOVA. ^*^Statistical significance was assessed relative to wild type (*p* < 0.05).

The ability of each strain to infect ticks was assessed using laboratory-reared *Ixodes scapularis* larvae. The larvae were infected by immersion in high density cultures of each strain, washed and then tested for spirochetes using PCR. All strains were able to cause at least a transient infection in ticks, indicating that PlzA and PlzB are not strictly required for early survival in the larval ticks (Table 4). Note that long term survival in ticks was not assessed in this study. Infected larval ticks were then fed on näive mice to determine if PlzA is required for spirochete transmission from ticks to mice. Infection status of the mice was assessed by serological analyses (ELISA and immunoblot) (Figure 5). All strains except B31-Δ*plzA* and B31-*plzB* KI were successfully transmitted from ticks to mammals as evidence by an antibody response. It should be noted, however, that only 1 of 3 mice that were exposed to ticks carrying B31-*plzA* KI developed antibody titers equivalent to that of the parental strain. The basis for the decreased efficiency of transmission of this strain is unknown. Immunoblot analyses using the pooled serum demonstrated that an antibody response to numerous proteins developed. Interestingly, there were significant differences in the array of proteins that were detected by infection-induced antibody in mice that were needle inoculated versus those that were infected by transmission from ticks (compare Figures 4B, 5B). Most notably, the mice infected by needle inoculation developed antibody against proteins in the 30–35 kDa range whereas the mice infected by tick feeding did not. Although not directly assessed, this difference most likely reflects differing antibody responses to OspA and OspB. It is firmly established in the literature that OspA and B are expressed at high levels in spirochetes cultured *in vitro* but not by spirochetes that transit into mammals from ticks. It can be concluded that PlzA is not strictly required to establish infection in ticks but is required to either allow spirochetes to transit from ticks to mice or to survive upon entering the mammalian environment. Importantly, these analyses also suggest that PlzB cannot substitute for or complement functions lost as a result of deletion of *plzA*.

**Table 4:** Tick to mouse transmission analysis.

**Strain**	**No. of ticks positive/tested**	**No. of mice culture positive/tested**
B31-wt	10/10	3/3
B31-Δ*plzA*	8/10	0/3
B31-*plzA* KI	8/10	1/3
B31-*plzB* KI	10/10	0/3

## Discussion

Cyclic di-nucleotides (CDN) are secondary messenger molecules that contribute to the regulation of a wide range of biological processes in bacteria (Jenal et al., 2018). Of these, c-di-GMP, which was discovered nearly 40 years ago (Ross et al., 1987), has been the most intensively studied. While there is considerable information about the function, domain organization and structure of DGCs and PDEs that produce and degrade c-di-GMP, respectively (Galperin et al., 2001; Jenal and Malone, 2006; Stock, 2007; Sondermann et al., 2012), the effector mechanisms of c-di-GMP in *Borrelia* are only partially understood (reviewed in Novak et al., 2014). In other bacteria, c-di-GMP exerts its regulatory activities through multiple mechanisms including direct binding to RNA riboswitches, transcriptional regulators, and a functionally and architecturally diverse group of proteins that harbor PilZ domains (Amikam and Galperin, 2006; Jenal and Malone, 2006; Römling and Amikam, 2006; Benach et al., 2007; Hengge, 2009; Jenal et al., 2018). The LD and relapsing fever spirochetes encode only a single known c-di-GMP receptor; PlzA and PlzC, respectively (Mallory et al., 2016). The dearth of c-di-GMP receptors in *Borrelia* is atypical, as most bacteria produce multiple c-di-GMP receptors. Earlier studies revealed that a subset of LD spirochete isolates carry a gene encoding a second PilZ domain protein designated as PlzB (Freedman et al., 2009). BLAST analyses of available genome sequences revealed limited distribution of *plzB* among Lyme disease isolates, which may reflect its location on a linear plasmid that is not essential for survival in ticks or mammals.

The ability of PlzB to bind c-di-GMP was assessed through nucleotide binding assays. Competitive binding experiments revealed that PlzB binds c-di-GMP in a specific manner (Figure 1). C-di-GMP binding was not inhibited by GTP, GMP, ATP, cGMP, or cAMP. PlzA and PlzC have been demonstrated to undergo significant structural rearrangement upon binding c-di-GMP (Mallory et al., 2016). It remains to be determined if a similar ligand-induced structural rearrangement occurs with PlzB. It can be concluded that PlzB binds c-di-GMP with high specificity and has the potential to serve as a c-di-GMP receptor *in vivo*.

To determine if PlzA and PlzB have distinct or synonymous functions, gene deletion and allelic exchange replacement studies were conducted in the *B. burgdorferi* strain B31 genetic background (see Figure 2). A *plzA* gene deletion mutant was generated by replacing the chromosomally encoded gene with a *strep*^*R*^ cassette (B31-Δ*plzA*). This strain served as the parental strain for subsequent genetic manipulations. *plzA-kan*^*R*^ and *plzB-kan*^*R*^ cassettes were used to replace the *strep*^*R*^ cassette of B31-Δ*plzA* to yield B31-*plzA* KI and B31-*plzB* KI. The *plzB* gene originated from *B. burgdorferi* ZS7. RT-PCR analyses confirmed that *plzB* is transcribed in the B31-*plzB* KI strain. As expected, *plzB* transcript was not detected in strains that lack *plzB*. Efforts to demonstrate PlzB protein production using immunoblot or IFA were inconclusive. This may be due to low level *plzB* expression. Consistent with this, earlier studies were unable to detect PlzA during *in vitro* cultivation (Freedman et al., 2009; Pitzer et al., 2011; He et al., 2014). In addition to low level expression, PlzA may also be compartmentalized in cells, potentially near the flagellar motors.

Interruption of c-di-GMP pathways, or an imbalance in its synthesis and degradation, has been demonstrated to result in aberrant motility phenotypes of *B. burgdorferi* (Freedman et al., 2009; Rogers et al., 2009; Caimano et al., 2011; Kostick et al., 2011; Pitzer et al., 2011; Sultan et al., 2011). Here the impact of genetic manipulation on both rotational and translational motility was assessed (results are summarized in Table 2). Strains were grown in BSK with or without 1% CMC. CMC increases the viscosity of the media and thus simulates the dense matrices spirochetes encounter in ticks and mammals. Rotational motility in BSK with or without CMC was not affected by deletion of *plzA*, indicating that PlzA is not required for the flagellar motors to function. Interestingly, relative to B31-wt and B31-*plzA* KI; B31Δ*plzA* and B31-*plzB* KI had significantly reduced translational movement with a significant decrease in flex frequency in BSK with CMC. Flexing is an inherent aspect of spirochetal motility that occurs when the flagellar motors located at opposite ends of the cell transiently rotate in the same direction. DIC microscopy revealed that the translational rate of B31-Δ*plzA* and B31-*plzB* KI was only 40% of that of the wt and B31-*plzA* KI complemented strains.

B31-Δ*plzA* and B31-*plzB* KI also displayed significantly reduced motility in swarm plate assays (Figure 3). Note that in the *Borrelia* field, the term “swarm assay” has been widely used to describe an assay that measures migration distance from a point of inoculation in a semi-solid media plate (i.e., colony size). To be clear, this is distinctly different from the phenomenon of “swarm motility” that has been described for some other bacteria including *Caulobacter* (Aldridge et al., 2003). We observed a significant decrease in colony size for the *plzA* deletion mutant (relative to wild type). Normal colony size was restored by complementation with *plzA* but not *plzB*. Pitzer et al also reported a similar level of reduced swarming. It is important to note that they also reported a change in colony morphology with the mutant and parental strains forming opaque and translucent colonies, respectively. The variable appearance of the colonies was concluded to represent phase variation. However, we saw no evidence of a distinct colony morphology for the B31-Δ*plzA* or B31-*plzB* KI strains. This discrepancy may reflect a simple difference in interpretation. While we observed denser “colonies” at the plate inoculation site, we suggest that this simply reflects a concentration of cells as would be expected based on their attenuated motility. Furthermore, Pitzer et al. did not demonstrate changes in protein expression that might be indicative of phase variation. While the overall findings of this report and the Pitzer study are in good agreement, there are subtle differences (Pitzer et al., 2011). It is possible that this is due to differences in the wt and parental strains used in each study. Pitzer used a clone derived from B31, designated as B31-A3-68, that has been engineered to have increased transformation efficiency. In this clone, ORF bbe02 was replaced with a *PflgB-aadA* cassette. This strain also lacks linear plasmid 56 and circular plasmid 9. The B31 derived clone (5A4) used here has not been subject to previous genetic manipulation and thus harbors bbe02. It is possible parental strain differences could be a contributing factor to the differences noted in Δ*plzA* mutant phenotypes between these two studies. We conclude that yet to be identified aspects of flagellar motor function are controlled directly or indirectly, at least in part, by PlzA and that PlzB cannot complement these functions.

Here we also investigated chemotaxis, as this represents another cellular activity that is dependent on controlled and directed motility (Figure 3C). Earlier studies demonstrated that c-di-GMP positively regulates the transcription of several genes involved in chemotaxis and NAG metabolism (Rogers et al., 2009; Kostick et al., 2011). Consistent with this, strains deficient in c-di-GMP production displayed reduced chemotaxis toward N-acetyl glucosamine (NAG). However, deletion of *plzA* did not result in a statistically significant difference in chemotaxis. While it is possible that chemotaxis to other chemoattractants could be affected, in this study we focused solely on NAG, a chemoattractant that is abundant in ticks.

Infectivity studies in C3H-HeJ mice using needle delivered spirochetes revealed that B31Δ*plzA* was non-infectious at a dose of 10^4^ spirochetes (Figure 4). This observation differs slightly from an earlier report that demonstrated that the infectivity of *plzA* deletion mutant was attenuated with an ID50 of 10^4^ (Pitzer et al., 2011). *Cis*-complementation of the B31Δ*plzA* strain in both this and the Pitzer study restored infectivity. We also sought to determine if the loss of PlzA function could be restored by *cis*-complementation of B31Δ*plzA* with *plzB*. As discussed above, while *plzB* transcription was demonstrated in the complemented strain, the ability to infect mice was not. All other strains infected mice and efficiently disseminated to distal tissues and organs. It can be concluded that PlzA is an important virulence factor required for *B. burgdorferi* to efficiently infect mice when delivered by needle inoculation.

The ability to infect mice when delivered by tick bite was also assessed (Figure 5). Since B31Δ*plzA* and B31-*plzB* KI were not able to infect mice when delivered by needle inoculation, ticks could not be infected by feeding larvae on mice. Hence, an “immersion feeding” approach was applied (Policastro and Schwan, 2003). This approach has proven effective and serves as a surrogate approach for studying the transmission process of strains that are not-infectious in mice (Kostick et al., 2011; Pitzer et al., 2011). The efficiency of infecting ticks by this approach was determined by PCR analysis of a random sampling of ticks to be >80%. All strains, including the mutant and complemented strains, infected ticks with similar efficiency. Pitzer also reported that a *plzA* gene deletion mutant could infect ticks. They also noted that the spirochete burden was significant reduced by day 7 post-immersion (Pitzer et al., 2011). In this report, we did not specifically assess the long-term survival of each strain in ticks. To assess transmission from ticks, 10 larval ticks were allowed to feed to repletion on mice. It is important to note in this report only 1 of 3 mice became infected with the complemented strain. It is unclear if this represents reduced infectivity or simply is a consequence of the small group size used in this experiment. In any event, the successful complementation of the non-infectious *plzA* mutant suggests that the non-infectious phenotype of B31Δ*plzA* was not the result of an unrelated secondary event. Cultivation and serological assays revealed B31-Δ*plzA* to be non-infectious. The B31-*plzB* KI strain was also determined to be compromised in its ability to transit from ticks to mice and or establish infection in mice. This observation, coupled with the needle inoculation analyses detailed above, suggests that the functions of PlzA and PlzB are not synonymous.

PlzA has been demonstrated to have global regulatory activity through its interactions with BosR and RpoS (He et al., 2014). It has been generally assumed that c-di-GMP binding serves to activate PlzA and consistent with this, FRET based analyses have revealed that PlzA undergoes significant structural rearrangements upon binding c-di-GMP (Mallory et al., 2016). While c-di-GMP binding could potentially serve as an on-off switch for PlzA function, studies presented to date suggest that the story is more complex. First, it has been firmly established that *B. burgdorferi* Δ*rrp1* mutants, which lack the ability to produce c-di-GMP, are infectious in mice but not in ticks. Second, as demonstrated here and in earlier reports, PlzA is required to infect mammals but is not essential for infection of ticks. *plzA* transcription, like that of *rrp1*, is upregulated in fed ticks (Freedman et al., 2009; Rogers et al., 2009). Since PlzA is the only known c-di-GMP receptor of most *B. burgdorferi* isolates, the concurrent upregulation of *rrp1* and *plzA* in ticks is not unexpected and could point to PlzA having a c-di-GMP dependent function in ticks. However, the fact that an *rrp1*^−^*/plzA*^+^ strain can infect mice while an *rrp1*^+^*/plzA*^−^ strain cannot, suggests that the essential functions mediated by PlzA in mammals are not dependent on c-di-GMP. It is our hypothesis that PlzA is a multi-functional protein that can carry out c-di-GMP dependent and c-di-GMP independent functions in different environments (i.e., tick and mammal).

In conclusion, this study provides further insight into the contribution of c-di-GMP receptors in *Borrelia* biology in both the tick and mammalian environment. It is striking that so few receptors have been identified in the *Borrelia*. It is probable that additional receptors or c-di-GMP binding partners (riboswitches, transcription factors etc.) do in fact exist but have not been identified by the methods that have been employed thus far. One of the confounding factors that could complicate the identification of additional c-di-GMP receptors is the environmentally regulated differential expression of a large number of *Borrelia* genes. There may be potential receptors that are produced during infection but not produced during cultivation. Regarding the biological significance of PlzB, it is clear that PlzB is not strictly required for *Borrelia* survival in ticks, in mammals or in culture. The potential *in vivo* functions of PlzB and its biological significance remains to be determined. As suggested in an earlier study, it is possible that the presence of both genes in a cell might enhance fitness and adaptive responses (Kostick et al., 2011). Studies that directly address this hypothesis are currently underway.

## Author contributions

JK-D conducted most of the experiments described within. JI, JF, LS, and LO all conducted experiments that were essential for confirmation of data presented within. JD and RM conceptualized the study framework and wrote the manuscript. JI and LO critiqued the manuscript and provided edits.

### Conflict of interest statement

The handling Editor declared a past co-authorship with one of the authors RM. The remaining authors declare that the research was conducted in the absence of any commercial or financial relationships that could be construed as a potential conflict of interest.
